# Reshaping the Curriculum for Academy in Factory in Malaysia

**DOI:** 10.3389/fpsyg.2023.1120611

**Published:** 2023-04-04

**Authors:** Khairul Azhar Jamaludin, Suganti Ealangov, Siti Nur Haslinda Md Saleh, Nor’Aqilah Ahmad Zabidi, Norlidah Alias, Mohd Hanafi Mohd Yasin, Bity Salwana Alias

**Affiliations:** ^1^Faculty of Education, Universiti Kebangsaan Malaysia, Bangi, Selangor, Malaysia; ^2^Faculty of Education, Universiti Malaya, Kuala Lumpur, Malaysia

**Keywords:** learning factory, teaching factory, academy in factory, industry-based curriculum, employability skills

## Abstract

Academy in Factory (AiF) was recently introduced in Malaysia to cater to the talent development and education attainment for a future-ready workforce. Though ideally expected to be an effective initiative, a lack of a strong curriculum framework hinders its implementation. To date the literature on AiF is very limited. It was found that its vague definition and characteristics, non-harmonised interaction between industry and academia, and non-existing curriculum framework are among contributing factors to its ineffective implementation. Thus, this study is aimed at reshaping a curriculum framework that is responsive to its aims and objectives. Building upon an extensive review of literature on AiF, industry-based curriculum, employability and human capital development, and best practices in the international context, the proposed framework is hoped to serve as a reference to improve its current practice.

## 1. Introduction

Since it was first introduced in 1994, the Learning Factory has received enormous support from institutions and industry. A learning factory, in general, consists of an educational component, an instructional component, and a productive environment (Schallock et al., 2018; Abele, 2019; Umeda et al., 2022). This means that learning in the factory is based on experiential and problem-based learning. Similarly, this approach, which is known as Academy in Factory (AiF) has been recently introduced in 2022 in Malaysia. Malaysian Productivity Corporation (MPC) (2022a) stated that this initiative is to enhance collaboration between industry and the national education system, produce skilled workers, close the gap between demand and supply of skilled workers, enhance local talents, and reduce dependency on imported labour. At the moment, AiF focuses on four sectors: semiconductor, automotive, life sciences and medical technology, and chemistry and advanced materials (Malaysian Productivity Corporation (MPC), 2022a).

However, in current practice, AiF targets local students who have at least acquired basic literacy and numeracy skills and passed the AiF screening, especially from the group of students who struggled in mainstream education (Malaysian Productivity Corporation (MPC), 2022a). Arguably, the current industry, which is demanding and challenging, necessitates workers who are highly skilled and competent in order to remain relevant in the fast-paced industry. With such low requirements, it is difficult for AiF to remain competitive while also ensuring that the selected students are potentially placed in higher education.

The broad concept and definition of AiF, on the other hand, remain hazy. In the context of Malaysia, current literature is limited to the description by Malaysian Productivity Corporation (MPC) (2022a,b), in which AiF is defined as innovative collaboration between industries and the Ministry of Education Malaysia (MOE). AiF’s implementation will be hampered by such a broad definition and characterization. As a result, the lack of structured organisation and delivery impedes the effective development of employability skills among AiF students. Industry active involvement is to develop both technical knowledge and skills of the students (Malaysian Productivity Corporation (MPC), 2022a), and with limited guidance and monitoring from local authorities, namely the Department of Skills Development and the Ministry of Human Resources, the focus and training of AiF may be ineffective.

As its focus is to provide: “…two-pronged solution for consistent talent supply for the manufacturing industry’s growth and improvement of Malaysia’s education attainment performance” (Malaysian Productivity Corporation (MPC), 2022c), the collaboration between four manufacturing sectors, Technical and Vocational Education and Training (TVET) institutions, local government, and agencies require a strategic planning. However, a non-harmonised interaction between industry and relevant stakeholders is not new in the context of skills development in Malaysia. Previous studies have identified that this issue is one of the major contributors to the ineffective development of local skilled workers in various TVET institutions (Rasul et al., 2015; Bassah, 2022). Potentially, AiF will face a similar problem.

In terms of its implementation, AiF is still in its infancy. Arguably, the framework for an effective implementation is still unavailable. What is available is a broad policy approach for talent development—Supply, Upgrade, and Matching (Malaysian Productivity Corporation (MPC), 2022b), which highlights the importance of sustaining the resource supply, upgrading local talents, and matching the needs of the industry with the produced local talents. Considering du Plessis’s (2017) perspective on the needs of a learning factory framework, the learning factory should serve as the link between educational institutions and real-world experiences in order to prepare graduates for future employment. He views the framework as a decision-making instrument for implementing a learning factory effectively. Thus, the lack of the current curriculum framework of AiF is potentially a hindrance to its success. Given its poor implementation and curriculum framework, there is a need to reshape its current practices based on a thorough examination of the literature and best practices in the international context. Thus, the current research aims to propose a curriculum framework for reshaping the current curriculum for AiF. Specifically, this study is aimed at answering: What is the curriculum framework for reshaping effective implementation of the AiF in Malaysia?

## 2. Literature review

### 2.1. Development of AiF in Malaysia

As the name suggests, a learning environment called a “learning factory” allows learners to gain real-world experience while learning. There are three phases in the history of learning factory development. In the 1990s, new approaches to application-based learning were created (Neacsu et al., 2021). Similarly, Assad et al. (2020) have reported that the concept of a learning factory experienced three distinct waves: the first wave occurred between 1986 and 2004, followed by 2004 and 2011, and the third wave between 2011 and the present. The Learning Factory network is well-established worldwide, and its effectiveness has been consistently proven (Abele et al., 2019).

The learning factory concept has been implemented in manufacturing, automotive, pharmaceutical, and life sciences. Despite the diversity of learning factory implementations, manufacturing remains dominant and is led by the world’s largest corporations. This is because learning factories can fill skill gaps and improve employability skills such as technical knowledge, interpersonal skills, communication, and management abilities (Mohd Ghazali et al., 2019).

In Malaysia, the MPC presented the concept of an Academy in Factory (AiF) around 2021. The learning factory was first implemented in 2022 in the automotive industry by DRB-HICOM. Initially, MPC solely directed toward the Twelfth Malaysia Plan: Developing Future Talent. Learning a factory’s ability to produce a proficient workforce along with the needs of Industry 4.0 cannot be denied; therefore, it has sparked innovations in the approach. Thus, to address the issue of labour demand in the high-profile economic sector, the government, industry, and academic institutions have collaborated to create a novel solution known as AiF (MPC 2022a).

The main objective of AiF is to develop a skilled workforce with sufficient motivation to engage in the industry. Similar to the learning factories that have been implemented across the globe, AiF is believed to bridge the gap between labour market demand and supply. AiF-trained individuals were equipped with specialised skills and knowledge developed by the industry to meet its requirements (Bernama, 2022).

### 2.2. Overlapping concepts of AiF

#### 2.2.1. Teaching factory

Various concepts were implicitly and formally created during the early stage of learning factories’ discovery. Early definitions were primarily derived from accounts of how learning factories were used, but there has been a robust exchange of scientific ideas in recent years. For instance, Jorgensen et al. (1995) characterised learning factories as activity-based facilities designed to be used across the curriculum. Learning factories are examples of such facilities. The idea behind this explanation, offered within the engineering education framework, includes an interactive experience of both the process and the result of its realisation. This is a dynamic experience since the tasks the learners complete shape the environment’s structure and its occupants’ activity. Recent implementations of learning factories are mainly founded on the same assumptions as earlier works; however, the idea of education and training has been enlarged, and the significance of realistic factory environments and processes has been emphasised (Abele 2019). The concept of “learning factory” generally refers to transdisciplinary, hands-on engineering design activities with close linkages to and regular engagement with the corporate world. Learning factories should include both educational and instructional components, as well as a productivity-oriented environment. The term “learning” emphasises the importance of learning through experience. This implies that the methods and technologies utilised in the learning factory are developed from those operated in actual industrial settings.

Specifically, the Initiative on European Learning Factories (2013) has defined a learning factory as a learning environment in which procedures and technology are modelled on a real industrial site, allowing for a hands-on approach to product development. Learning factories are built on a pedagogic concept emphasising experiential and problem-based learning. Therefore, the idea of continuous development is fostered by the participation of the participants. Fatimah et al. (2019) have highlighted the idea that the learning factory has its roots in the medical sciences and, more specifically, in the paradigm of teaching hospitals, which strives to incorporate the learning and working environment from which genuine and applicable learning experiences develop. It also includes various teaching methodologies to bring the teaching and learning processes closer to real industrial challenges. In manufacturing, however, the term learning factory has been described as a learning environment with actual processes involving multiple stations and incorporating both technological and organisational elements in a virtual manufacturing environment. The learning factory consists of formal, informal, and non-formal learning that enables trainees to learn in an on-site learning environment (Ogorodnyk et al., 2017; Assad et al., 2020; Roll and Ifenthaler, 2021).

#### 2.2.2. Learning factory

The learning factory approach’s basis is the teaching factory, which integrates industries into the education system. Its objectives include working and learning environments that produce realistic and applicable learning experiences. The idea is that learners and staff “educate” manufacturing practitioners about advancements made in manufacturing technology, new trends, and the outcomes of research and development activities that happen from a classroom to a factory (Mavrikios et al., 2019).

According to Stavropoulos et al. (2018), the teaching factory aims to incorporate industrial partners and difficulties into the educational process, adopting a concept comparable to the learning factory. The idea behind the “teaching factory” is to gradually integrate engineers into the industrial field by using the production difficulties located on them. The teaching factory functions as a bi-directional knowledge communication channel, “bringing” the actual factory to the classroom and the academic to the factory. It is a non-geographically anchored learning “space” facilitated by advanced ICTs and high-grade industrial didactic equipment. A practice-based curriculum and advanced manufacturing equipment are integrated into the learning factory.

While Sumual and Soputan (2018) stated that the teaching factory allows learners to master psychomotor, cognitive, and affective standards of mastery while raising the outcome of learning-inspired and intuitive behaviour, referred to academically as character learning. Three main reasons support the concept of a teaching factory: (1) traditional education is insufficient; (2) learning benefits from hands-on experience; and (3) team-based learning activities involving students, faculty, and industry participants enrich the educational process and offer real advantages. Thus, it can be concluded that a teaching factory is a learning activity in which learners actively produce goods or services while still in the context of a formal academic environment.

#### 2.2.3. Apprenticeship

The labour force is a resource for the industry sector and plays a crucial role in the performance of projects. The shortage of workers is associated with low profits for contractors and poor project performance (Construction Industry Development Board, 2017). Thus, recruiting young people is vital for the long-term sustenance of the construction sector (Daniel et al., 2020).

Apprenticeship programs were designed to appeal to young people, and at the end of the training, learners gain the skills necessary to perform a trade. These training programmes are essential for sustainable talent management and raising labour productivity (Gardas et al., 2019). Apprentice training programmes that were completed would make it easier to replace the ageing workforce and enhance the achievements of construction projects.

According to Cunningham (2021), an apprentice has committed to labour for a specified period at low pay while learning a trade from a skilled employer. The Middle Ages are where the word “apprentice” first appeared. It derived from the old French “aprentis” from the verb apprendre, which means “to study.” Ryan and Unwin (2001) define apprenticeships as a structured programme of vocational preparation sponsored by an employer and compare part-time education with on-the-job training and work experience. It led to a recognised vocational qualification at a craft or higher level; a more modern definition may be more in line with professional apprenticeships.

#### 2.2.4. Work-based learning

Work-based learning is an effective teaching-based learning strategy used in vocational schools (Ferrández-Berrueco et al., 2016). It is an educational activity intended to combine classroom instruction with industry activities.

The internship is a work-based learning strategy that allows students to work in the industry for a predetermined amount of time (Cooper et al., 2010; McHugh, 2016). This practice is well-known as “industrial work training” at certain vocational schools. Through internship experiences, instructors want learners to be able to put the knowledge they have acquired in the curriculum into practice in business and industry. In other words, after completing the internship programme, students should begin getting ready to work in their area of expertise (Prianto and Qomariyah, 2020).

UNESCO (2021) stated that any learning strategy in a real-world working environment is known as work-based learning. It allows people to advance their professional development to obtain and keep jobs. Thus, work-based learning makes integration possible because it provides learners with the skills that employers need in the workplace.

### 2.3. Redefining AiF

Although there is no clear scientific definition of AiF in the literature, the idea emphasised by AiF is similar to that of a “learning factory,” because both statements are identical in their approach. Generally, AiF can be interpreted as an active learning environment in the industry on a hands-on basis that adheres to the concept of “working while learning” to create a trained workforce that can meet industry demands and the labour market’s requirements. This means that the designated industry provides the identified apprentices with 18 months of training in general and specialised factory operations (Malaysian Productivity Corporation (MPC), 2022a,b).

At the moment, Malaysian Productivity Corporation’s (MPC) (2022a,b) definition which describes AiF as innovative collaboration between industries, and the MOE is arguably comprehensive. For a better understanding, a more comprehensive definition is required. Revisiting other literature in the international context [such as by Jorgensen et al. (1995); Stavropoulos et al., 2018, and Sumual and Soputan (2018)], AiF should be characterised as an innovative effort to:

1. Encourage interactive experience of academic and work-based activities;
2. Expose students to transdisciplinary and hands-on tasks in a productivity-based environment;
3. Integrate relevant technologies to support learning and skills development; and
4. Encourage team-based learning between students, industry, and academia.

Given these problems, the above definition, and characteristics of AiF are important to make sure that not only policymakers, but also people in industry and academia, have a clear idea of what AiF is and how to make it work.

### 2.4. Learning from the best practices of the learning factory

Globally, numerous approaches, strategies, and models have been applied to learning factory. Numerous industrial nations, including Germany, Austria, and other European nations, have implemented learning factories to increase industrial productivity. Even Asian nations, such as China and Japan, have created learning factories to maximise local talent for economic growth. In Japan, learning factory is an approach to elaborate the engineering process in a structured manner to develop skill workers (Umeda et al., 2022). Schallock et al. (2018) found that the implementation of the learning factory prioritises the development of technical, transformational, and social skills, regardless of the approaches and models used. In Germany, for instance, these skills are developed by facilitating interaction between trainees and instructors and by maximising their interactions in factory settings (Schallock et al., 2018).

According to De Zan et al. (2015), there are four phases of experiential learning in the learning factory, as determined by a closer examination of the current practise. The objective of the first phase is to develop concrete experiences by providing an introductory briefing and exploration opportunities. In either model factories or actual factories, trainees will gain practical experience in a simulated setting (Abele and Eichhorn, 2008). According to Engeström and Sannino (2010), during this phase of learning, trainees’ opinions and ways of thinking are challenged. Following this is the reflective observation phase. This implies that both trainees and their organisation will continuously share and re-expound on their experiences throughout this process. The third phase involves abstract conceptualization, which is the application of theoretical concepts in explaining and organising the planning. Thus, the trainees will abstractly generalise their experiences, while their trainers will explain and direct this process. Trainers correct any errors made by trainees and use this explanation as a link for subsequent learning processes. The concluding phase is experimentation, which consists of state application and consolidation. During this phase, the trainees will implement the planned activities while their trainer configures the model factory. While the consolidation procedure is a process where they must reflect past actions for proposing recommendations and improvements (Engeström and Sannino, 2010).

Enke et al. (2017) and Tisch and Metternich (2017), on the other hand, have proposed a didactic concept with a psychological approach to achieving a successful learning factory process. Enke et al. (2017) have designed a competency-oriented development of learning factories with three learning levels: macro, meso, and micro. As a foundation for learning, learning objectives must be clarified and defined at the macro level. While the meso level requires designing a teaching module with sub-competencies and sequences, the micro level requires designing specific teaching-learning situations (Tisch et al., 2016).

In a recent study by Wienbruch et al. (2018), the didactic concept was proposed to form a new learning factory module for enhancing learning factories. In the initial phase, trainees were introduced to Industry 4.0 fundamentals. The second phase of the module introduces trainees to the implementation’s tools and techniques. In the third phase, the trainee-identified objectives from the first and second phases are determined. After the third phase of implementation, phases 4 through 7 consist of the evaluation process, which includes audit, maturity model, measures deduction, and evaluation. After the evaluation phase, the factory learning module is described during the iteration phase. Moreover, the process and outcome are reflected once more in the final phase.

### 2.5. Challenges to effective implementation

The learning factory implementation would fail if the current issues with learning factory approaches were not addressed. Leal et al. (2020) and Li et al. (2019) both agreed that traditional ways of teaching in learning factories were not keeping up with how technology was changing. As a result, the use of technology did not support and maximise teaching practises. Specifically, the comprehensiveness of the practical training content, insufficient training of students’ consciousness, and teaching content and methods that are still not responsive to technological advancements.The content and environments of typical learning factories, according to Enke et al. (2017), were designed by technical experts from industry who were more concerned with the working processes than the development of knowledge and competencies. Furthermore, the learning modules were created with ambitious learning objectives.

Sumual and Soputan (2018), on the other hand, identified a lack of equipment and facilities provided by educational institutions or industries as a challenge to implementing an effective learning factory. This is support by Mourtzis et al. (2018) and Mavrikios et al. (2019) said the expensiveness of the equipment, which enforces the use of outdated equipment, is a drawback of this approach. The study by Tisch et al. (2016) showed that uncertain pilot situation is caused by no systematic approaches on learning factory design. Lack of the objectives of the courses also can lead to the poor practices of learning factory. In addition, lack of reliable instruments to evaluate the development of intended competencies in learning factories will affect the outcome (Tisch et al., 2016).

### 2.6. Underpinning concepts and theory to talent development

#### 2.6.1. Employability skills

To improve a worker’s work abilities, employability skills are required. Employees at all levels should be able to adapt to changes in job requirements, work conditions, and obstacles in the global labour market if they have employable skills. The term “employability skills” refers to a set of abilities that can be applied to a variety of jobs. These abilities are important because they provide people with the knowledge, skills, and attitudes required to work in the 21st century (SCANS, 1991). Alternatively, Okolie et al. (2020) define employability skills as a set of qualities that, when developed, make graduates more likely to find jobs in their selected sectors. Additionally, Sarkar et al. (2019) defined employability skills as a combination of generic and discipline-specific skill sets, as well as career management abilities. Outside of the context of schooling, generic skills may be used for a range of jobs. These attributes are also known as soft skills, core talents, transferrable abilities, and important competencies.

Interpersonal skills, intrapersonal skills, and information, communication, and technology (ICT) skills are the most generic employability skills that are widely applicable across most jobs (Humburg and Van der Velden, 2017; Sarkar et al., 2019). According to Nadarajah (2021), Malaysian graduates are unemployed not because of a lack of employability skills, but because of a mismatch between labour market expectations and local graduates. As the workforce changes, employers face significant challenges in the fiercely competitive global market. The new economy necessitates workers with new skill sets, which must be met through education and training. As a result, educational and industrial training institutes should educate trainees in order to maximise their potential and meet the demands of employers for employability skills. Perhaps there is an urgent need to include employability skills in the training programme.

#### 2.6.2. Human capital development

Human capital development is another term for human-centred development. The primary goal is to broaden and deepen relationships with individuals in order to secure future economic benefits. Fundamentally, it refers to an individual’s level of expertise and dedication in the context of an organisation, and it is measured in terms of their experience, potential, and capacity. Health facilities, on-the-job training, education, adult education, and migration, according to Schultz (1971), are five categories that can be focused on enhancing human capacity and producing human capital. All of this is not formed naturally or through work experience alone, but rather through a lengthy, time-consuming education and training programme that allows an individual to be skilled in his or her job. According to Sima et al. (2020), the intrinsic framework for the Industry 4.0 is the growth of human capital and an individual’s creativity. Human capital may play a significant role in the work and be redirected in terms of jobs and learning. As a result, it is critical to adapt the education system to meet the present social growth requirements. However, the situation in Malaysia remains unfavourable in terms of industry involvement in skill development and human capital development (Adam et al., 2020). In order to meet the industry’s current needs, AiF programmes must consider human capital development.

#### 2.6.3. Cognitive apprenticeship theory

Cognitive apprenticeship theory (CAT) is an instructional strategy based on the contextual learning paradigm. The apprentice master model of traditional crafts, which is common in non-formal educational settings, served as the foundation for this concept (Lo and Tsai, 2022). It has, however, been adapted to apply to “cognitive” or “intellectual” domains. In Brown et al. (1989) proposed that the idea of teaching students in schools using a system similar to that of an apprenticeship in a skill. Cognitive apprenticeship is a progressively guided learning process that makes use of expert models and feedback from experts. One of the most significant differences between the two is how the CAT approaches task visibility in comparison to the traditional model. Traditional apprenticeship is a limited instructional paradigm for effectively transmitting higher-order problem-solving abilities or tacit meta-cognitive information. This is due to the fact that higher-order problem-solving abilities and metacognitive knowledge are both implicit. It is possible to observe the candidate actually performing the job or skill during the CAT. Similarly, a focus of these two models is on thinking that must precede and be a component of the work, as well as follow any necessary observations made after its completion. The CAT employs six primary learning schemes during learning activities: modelling, coaching, scaffolding, articulation, reflection, and exploration (Matsuo and Tsukube, 2020). In recent years, CAT has received widespread attention as a potential instructional approach for improving trainees’ higher-order cognitive abilities. As a result, the AiF programme must be based on this theory in order to improve the cognitive skills of the participants.

#### 2.6.4. Industry-based curriculum

Because the current education system is unable to meet the demands of the industry, employment training programmes must incorporate an industry-based curriculum (Jamaludin et al., 2021). This mismatch leads to high unemployment, which tarnishes education’s image in the eyes of the public, who begin to question the system’s efficiency. The shifts brought about by Industry 4.0 in terms of the employment environment have prompted the requirement for new skills that are in line with the development of advanced technology. Therefore, graduates must be exposed to the releavnt skills that correspond to these breakthroughs to equip them for the challenges of Industry 4.0. (Rodzalan et al., 2022).

To address this issue, educational institutions and industries must work together to provide real-world work experiences and training. Similarly, the industry should be involved in the design of the AiF program’s partnership programme from the start. The program’s development, expansion, and management must receive a significant amount of focus and attention. The industry, in particular, must be given the opportunity to participate in the process of developing industry-based curricula and assessing training. To avoid the training content becoming obsolete, ensure that the industry’s demands are represented in the training content. Furthermore, the design of curriculums must be adaptable and based on a labour-market-oriented approach. This approach aims to develop workers who are capable of learning new skills on the job. An industry-based curriculum, according to Wellington (1993), is distinguished by the incorporation of work-based competencies into the national curriculum, the development of an appropriate framework, personal and social education programmes, and the inclusion of work-related activities. These are the four general characteristics of a curriculum based on industry. Industry participation is critical because one of the goals of an industry-based curriculum is to produce workers who are knowledgeable and skilled. As a result, increasing industrial participation in teaching and learning is critical.

#### 2.6.5. Taba’s curriculum planning model

Taba’s curriculum planning paradigm allows for flexibility in curriculum design. It enables curriculum designers to identify specific learning outcomes, which can then be matched up with the corresponding assessments of those objectives. These parameters are ideal for the industrial-based AiF programme. According to Riafadilah and Mukhidin (2018), a curriculum can be thought of as a learning plan; thus, what is understood about learning and personal growth influences curriculum design. Taba recommends using an inductive approach to curriculum design. According to Taba, the process of developing a curriculum can be divided into seven steps: identifying student needs, deciding on curriculum goals, selecting curriculum content, organising the content, selecting learning experiences, and evaluating the curriculum (Yusof et al., 2018). Curriculum design is an ongoing process, and instructional and curriculum committees will play critical roles in shaping the curriculum through critical criticism. This is due to the involvement of academia and industry in the development of the AiF curriculum.

#### 2.6.6. Malaysian skills certification

The AiF programme gives industries an unprecedented opportunity to drive their own skills agenda and generate training opportunities for early career entrants and mature learners; however, there is significant concern when employers are hesitant to obtain adequate accreditation and certification, casting doubt on the program’s efficacy. Accreditation ensures that the curriculum meets or exceeds the educational and industrial criteria outlined in national standards. The Malaysian Skills Certification Framework highlights the types of competencies required for producing skilled and knowledgeable individuals in accordance with the National Occupational Skills Standard in the Malaysian context (NOSS). This standard’s goal is to ensure that individuals acquire the necessary aptitude and motivation to succeed as skilled workers by participating in excellent educational opportunities. The accreditation policy provides a method for evaluating training programmes that are not part of the mainstream educational system. Furthermore, it addresses the issues raised by technological advancements by encouraging continuous curriculum development through a continuous evaluation process. From the trainee’s point of view, obtaining an approved qualification is most likely their primary goal. This is done primarily to aid in professional development and career advancement, but it is also done to stay current and prevent skills from becoming obsolete. As a result, it stands to reason that a programme lacking adequate certification and accreditation will have an impact on the motivation of the participants. As a result, in order to obtain Malaysia Skills Certification, the industry must provide training that meets the National Occupational Skills Standard (NOSS). This will increase both the industry’s and trainees’ motivation.

## 3. Reshaping the current AiF curriculum in Malaysia

**Figure 1:**
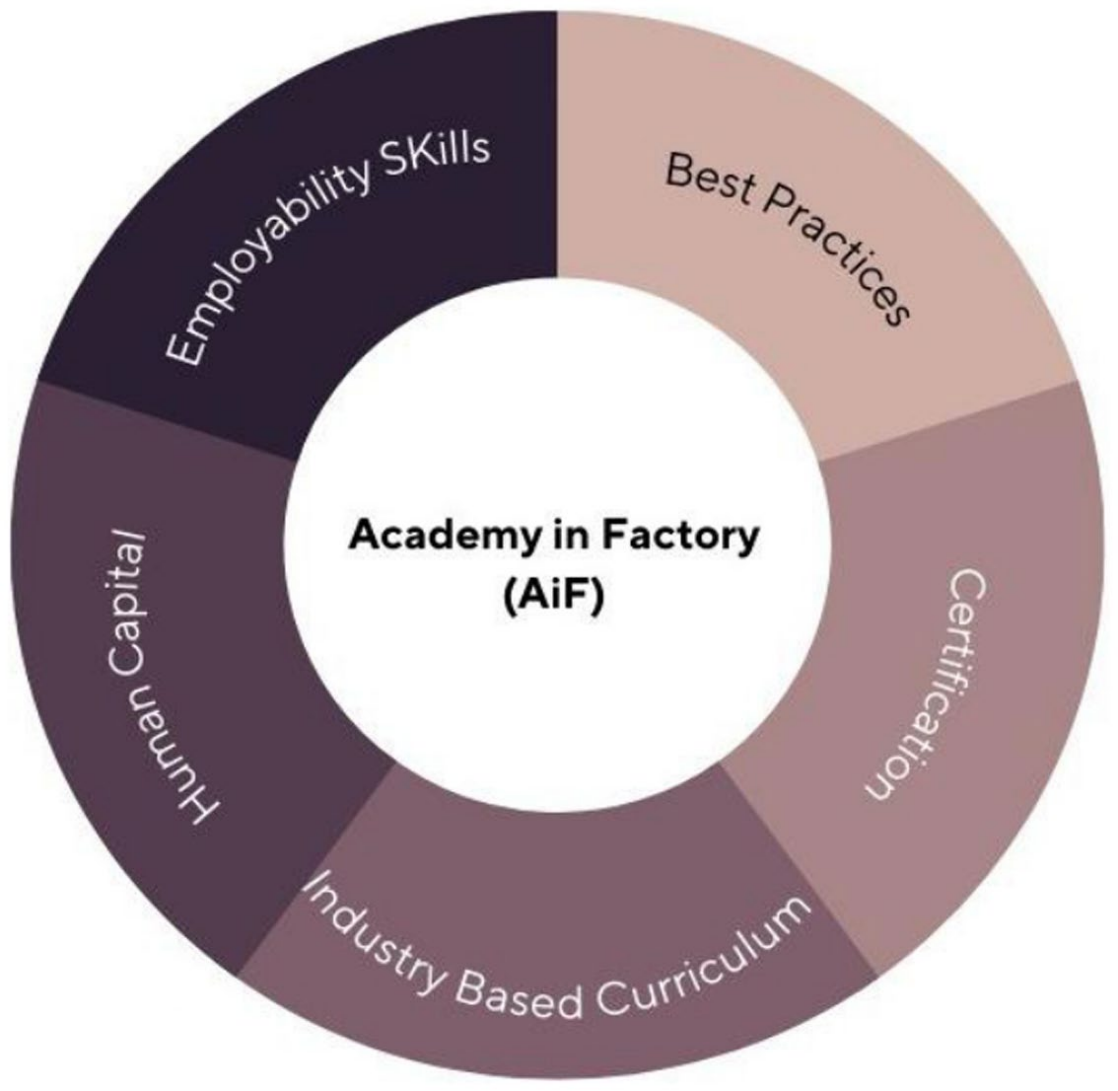
Framework for Malaysian AiF.

The current AiF programme is not being implemented consistently across industries. As a result, a common curriculum framework that brings together industry, academia, and government is urgently needed. A clear direction is required to ensure that the objectives of the industry implementing this programme and the goals of policymakers do not conflict. Despite the fact that the AiF programme is still in its early stages of implementation, a number of issues have been identified, including a lack of a clear definition, a lack of implementation instructions, and enrollment and recognition issues. As a first step, this study recommends to stakeholders a framework based on five key components: industry-based curriculum, employability skills, human capital, best practises, and certification. Figure 1 depicts how this framework is generally conceived.

In Malaysia, the AiF programme is still in its infancy, but it has many parallels with Learning Factory, teaching factory, apprenticeship and work-based learning. The fundamental purpose of each of these concepts is identical. The majority of nations have successfully adopted AiF according to their respective models, such as Germany’s Learning Factory, and Austria’s Learning Factory. Therefore, these best practices can be accommodated in Malaysia. For example, the implementation in Germany, Austria, and other European countries should serve as the primary benchmark for improvising AiF procedures. Redesigning the AiF should be based on implementation models by Wienbruch et al. (2018), Tisch et al. (2016), and Karre et al. (2019). Consideration should be given to reshaping the current AiF curriculum to incorporate didactic concepts and experiential learning phases. It is believed that experiential learning facilitates the development of a solid foundation of knowledge and concrete industry experiences (Enke et al., 2017; Tisch and Metternich, 2017). To support the didactic concept of learning factories, trainers and curriculum developers must precisely identify relevant training objectives and develop modules, as well as create authentic situations in which trainees can apply their theoretical knowledge in practise (Tisch et al., 2016; Wienbruch et al., 2018).

In addition, the CAT (Collins et al., 1989) and the Taba’s curriculum planning model (Taba, 1962) may be utilised to reframe the programme. This means that the objectives, learning content, delivery method, and assessment method should be parallel with the requirements in the Malaysian Skills Certification. Also, to be relevant to the current industry, the principles of the industry-based curriculum should be considered, especially industry involvement in designing work-related activities and skills development (Wellington, 1993). As highlighted by Jamaludin et al. (2021), components in the industry-based curriculum should be utilised in reshaping the foundation of AiF. This includes a closer identification of relevant skills that are not only limited to a specific factory that the trainees are attached to, but to consider the development of other employability skills and national curriculum (such as the TVET curriculum p.

Furthermore, the proposed framework included employability skills and the need to develop human capital to achieve AiF objective. This means that the focus of skills development should not only limited to the needs of the industry but also the employability skills that focus on the 21st century skills as proposed by SCANS (1991) that are relevant to human capital development. The framework is therefore help to address the skills gap, which will be achieved by bringing together industry-academic partnerships through industry-based curricula.

In addition, the certification element based on the NOSS standard is recommended in order to improve the quality of AiF implementation and guarantee the program’s continued relevance. This necessitates that the outcomes of this training be aligned with the Malaysian Skills Certificate, which is commonly utilised in Malaysian TVET institutions. MPC (2022a) stated that the AiF certificate (equivalent to Level 3 Malaysian Skills Certificate) is awarded 18 months after completion.

In short, the proposed framework for reshaping the current AiF is believed to have the potential to further strengthen and address issues with its poor concept and implementation, thereby assisting the AiFs in achieving their goals of providing skilled workers and talent development to individuals at national level (aged 18–35) in the semiconductor, automotive, life sciences and medical technology, and chemistry and advanced materials industries (MPC, 2022b).

## 4. Conclusion

A closer analysis of the current literature and practices has yielded a number of intriguing arguments. It was found that the AiF definition and characteristics are still vague, thus hampering a general understanding of its implementation. Also, the current practice lacks a strong foundation, especially in its curriculum planning and implementation. This paper has addressed these issues by proposing a framework to reshape the current AiF curriculum. The proposed framework is built upon the model of industry-based curriculum, employability skills, human capital, best practices of learning factories at the international context, and certification that is based on the NOSS and Malaysian Skills Certificate Standards.

As a result, the proposed framework is useful in guiding ministries, particularly education and human resource development, curriculum developers, industry, and TVET institutions, in improving current AiF practise. Instructors, trainers, and industry participants, in particular, should be equipped with a solid foundation of AiF knowledge and understanding to help them understand an effective way to provide training and improve trainees’ employability skills. This is due to the fact that their skill development is dependent on the competency of the trainers (Mei Kin et al., 2022). On the other hand, the rise of Industrial Revolution 4.0 necessitates that training programmes be responsive to the needs of the learners (Abdullah et al., 2022; Alam et al., 2022); therefore, the proposed framework provides a clear direction to assist AiF training in becoming more effective and responsive to the industry’s current needs.

However, it is suggested that future researchers develop a specific curriculum for the effective implementation of AiF in Malaysia. This includes involving local industry experts, academicians, stakeholders, and other relevant bodies in TVET to develop a sound curriculum for AiF based on the current findings. Furthermore, an investigation of the current and revised AiF curriculum in Malaysia is critical to better understand its effectiveness and propose improvements for future implementation.

## Data availability statement

The original contributions presented in the study are included in the article/supplementary material, further inquiries can be directed to the corresponding author.

## Author contributions

KJ, SE, SS, and NZ: conceptualization, writing—review, and original draft preparation. KJ, NA, MY, and BA: review. All authors contributed to the article and approved the submitted version.

## Funding

This work was supported by Geran Galakan Penyelidik Muda (GGPM-2021-013).

## Conflict of interest

The authors declare that the research was conducted in the absence of any commercial or financial relationships that could be construed as a potential conflict of interest.

## Publisher’s note

All claims expressed in this article are solely those of the authors and do not necessarily represent those of their affiliated organizations, or those of the publisher, the editors and the reviewers. Any product that may be evaluated in this article, or claim that may be made by its manufacturer, is not guaranteed or endorsed by the publisher.
